# P2Y_12_ Inhibitor vs Aspirin Monotherapy Following Dual Antiplatelet Therapy after Percutaneous Coronary Intervention: An Updated Meta-Analysis

**DOI:** 10.31083/j.rcm2410284

**Published:** 2023-10-08

**Authors:** Tong Gao, Chang Meng, Yintang Wang, Siyuan Li, Lei Bi, Yu Geng, Ping Zhang

**Affiliations:** ^1^Department of Cardiology, Beijing Tsinghua Changgung Hospital, School of Clinical Medicine, Tsinghua University, 102218 Beijing, China; ^2^Department of Emergency, Emergency General Hospital, 100028 Beijing, China

**Keywords:** P2Y_12_ inhibitor, aspirin, ischemic heart disease, percutaneous coronary intervention

## Abstract

**Background:**

With the publication of a large number of clinical studies on antiplatelet therapy in recent years, it is still controversial which antiplatelet monotherapy should be continued after a period of dual antiplatelet therapy (DAPT) in the post percutaneous coronary intervention (post-PCI) population. We conducted a meta-analysis to investigate the efficacy and safety of ${\mathrm{P2Y}}_{12}$ inhibitors versus aspirin in the post-PCI population after completing DAPT.

**Methods:**

We searched studies in electronic databases from January 1, 2015 to November 20, 2022. We conducted a meta-analysis to estimate the effect of ${\mathrm{P2Y}}_{12}$ inhibitor monotherapy on clinical end-points in post-PCI patients after a period of DAPT, using trial-level data with consistent end-point definitions. The primary outcome was major adverse cardiovascular events (MACE). Odd ratio (OR) was pooled with 95% confidence interval (CI) for dichotomous data. This study is registered with INPLASY 2022120011.

**Results:**

We included five studies that included 24,460 patients. The patients who received a ${\mathrm{P2Y}}_{12}$ inhibitor showed a lower risk of MACE than patients who received aspirin (OR 0.70 [95% CI 0.60–0.80], $I^{2}$ = 0%, *p <* 0.00001) monotherapy. Subgroup analysis of MACE based on patient characteristics showed consistent results with the main analysis. The risk of major bleeding was similar in patients who received a ${\mathrm{P2Y}}_{12}$ inhibitor and those who received aspirin (OR 0.86 [95% CI 0.53–1.39], $I^{2}$ = 57%, *p* = 0.54). The risk of major bleeding was borderline increased in patients who received ticagrelor versus aspirin (OR 1.81 [95% CI 0.99–3.31], *p* = 0.05).

**Conclusions:**

In the post-PCI population, ${\mathrm{P2Y}}_{12}$ inhibitor monotherapy may be superior to aspirin for MACE, repeat revascularization, and stroke without increasing the risk of major bleeding.

## 1. Introduction

Ischemic heart disease is one of the most common cardiovascular diseases in the world, and percutaneous coronary intervention (PCI) is an effective means to treat it [1]. The number of PCI procedures is increasing year on year. According to current guidelines, using dual antiplatelet therapy (DAPT) consisting of aspirin and ${\mathrm{P2Y}}_{12}$ inhibitors after drug-eluting stent placement can reduce the risk of postoperative thrombotic complications [2, 3, 4]. The routine duration of DAPT in patients with chronic coronary syndrome (CCS) is 6 months. The routine duration of DAPT in patients with acute coronary syndrome (ACS) is 12 months [1, 2, 3, 4]. Following DAPT, single antiplatelet therapy (SAPT) is used for secondary prevention, and aspirin is generally used as the first choice due to positive results from previous randomized clinical trials [5].

Recently, consideration of the potential risk of aspirin-related gastrointestinal complications has prompted research into non-aspirin treatments following PCI [6]. Two studies demonstrated that clopidogrel showed similar clinical outcomes in patients after PCI compared to aspirin [7, 8]. Recent evidence suggests that SAPT with ${\mathrm{P2Y}}_{12}$ inhibitor is superior in balancing bleeding and ischemic risk [9, 10, 11]. An extended HOST-EXAM (Harmonizing Optimal Strategy for Treatment of Coronary Artery Stenosis–Extended Antiplatelet Monotherapy) study with more than 5 years of follow-up showed that clopidogrel monotherapy showed a lower rate of compound net clinical events in patients with no clinical events 12 ± 6 months after stent PCI compared to aspirin monotherapy [12]. A meta-analysis which included five clinical trials found that clopidogrel showed a lower major adverse cardiovascular events (MACE) and stroke rate after DAPT completion after PCI compared to aspirin, while there were no significant differences between the two groups in mortality, major bleeding, myocardial infarction, and repeated revascularization [13]. We know that ${\mathrm{P2Y}}_{12}$ platelet receptor inhibitors are not just clopidogrel. Most recently, an analysis of the GLOBAL LEADERS trial found that ticagrelor monotherapy showed a lower ischemic composite endpoint compared to aspirin monotherapy. In contrast, ticagrelor monotherapy showed a higher major bleeding endpoint [14]. It is still controversial which antiplatelet monotherapy should be continued after a period of DAPT in the post-PCI population. Therefore, an up-to-date and comprehensive analysis of this issue is necessary.

The aim of this meta-analysis was to bringing together data from all available prospective clinical studies investigating the efficacy and safety of ${\mathrm{P2Y}}_{12}$ inhibitors versus aspirin in the post-PCI population after completion of DAPT.

## 2. Methods 

Our current meta-analysis follows the performing and reporting specifications of the Preferred Reporting Items for Systematic Reviews and Meta Analyses (PRISMA) guidelines [15]. We registered the protocol on the International Platform of Registered Systematic Review and Meta-analysis Protocols database (Inplasy protocol: INPLASY 2022120011) and is available on inplasy.com (https://inplasy.com/inplasy-2022-12-0011). Our research did not require ethical approval.

### 2.1 Search Strategy 

Three independent researchers conducted an extensive electronic search of relevant articles published between January 1, 2015 and November 20, 2022. The database includes Embase, PubMed and the Cochrane database. We independently hand-selected relevant randomized controlled trials (RCTs) and screened any relevant studies. The literature search strategy is shown in **Supplementary Table 1**.

### 2.2 Inclusion and Exclusion 

Document management was performed using EndNote X9 version (Thomson Corporation, Stanford, CT, USA) software, and the eligibility of the identified items was independently evaluated by two investigators. First, the title and abstract were first screened. Eligible articles were retained for reading in full-text review. The inclusion criteria for eligible studies included: (1) Patients receiving dual antiplatelet therapy after PCI. (2) Treatment with ${\mathrm{P2Y}}_{12}$ inhibitor or aspirin monotherapy. (3) Outcome indicators: MACE, all-cause death, cardiac death, myocardial infarction, major bleeding, stent thrombosis, repeat revascularization and any stroke. The exclusion criteria include: (1) Clinical study of DAPT compared with SAPT. (2) Studies evaluating antithrombotic drugs other than aspirin or ${\mathrm{P2Y}}_{12}$ inhibitors. (3) There is not enough data to extract, such as abstracts of some meetings, literature reviews, pharmacological introductions, etc. (4) Retrospective studies were also excluded.

### 2.3 Bias & Quality Assessment

The three researchers independently evaluated, screened and examined the literature according to a unified and standardized method, and included the literature according to strict inclusion and exclusion criteria, and then conducted data collection and analysis. We evaluated the quality of the selected articles according to the quality evaluation criteria of the Newcastle-Ottawa Scale and Cochrane Reviewer Handbook 5.1.0 [16].

### 2.4 Data Synthesis and Analysis 

This meta analysis selected Revman 5.3 (The Nordic Cochrane Center, Copenhagen, Denmark) and Stata 14.0 (STATA Inc., College Station, TX, USA) for data management and analysis. The data which met homogeneity (*p* > 0.10 and $I^{2}$ ≤ 50%) through a heterogeneity test were meta-analyzed with a fixed effect model. If homogeneity (*p* ≤ 0.10 or $I^{2}$ > 50%) was not met, and heterogeneity could not be excluded, a random effects model was used to combine effects, but it should be noted that the type of data analyzed should consider sensitivity analysis and subgroup analysis. We merged the results from all relevant studies to estimate the pooled risk ratio (RR) and associated 95% confidence intervals (CIs) for dichotomous outcomes. Statistically significant was defined as *p* < 0.05.

## 3. Results

**Fig. 1.**
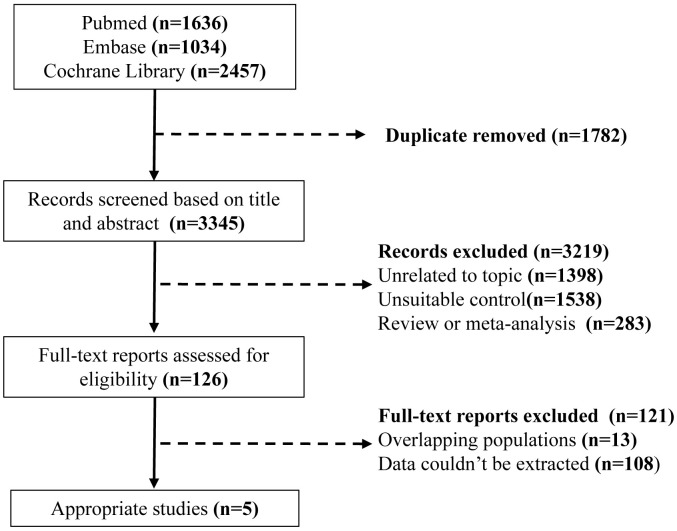
**The flow chart of study selection process**.

The search and research selection process is summarized in a flow chart (Fig. 1). Of the 5127 studies identified by electronic search, 1782 studies were excluded due to duplications. After reading the title and abstract, we excluded 3219 studies that did not meet the inclusion criteria. The remaining 126 studies were evaluated by reading the full text. Data from 5 trials evaluating ${\mathrm{P2Y}}_{12}$ inhibitor versus aspirin monotherapy after coronary stenting were included.

Table 1 (Ref. [7, 8, 11, 12, 14]) shows the main features of the included trials. In our analyses, a total of 24,460 patients were assigned to aspirin (n = 10,661) or ${\mathrm{P2Y}}_{12}$ inhibitor monotherapy (n = 13,799). All of the studies were on clopidogrel monotherapy following dual antiplatelet therapy after coronary stenting except for the trial reported by Masafumi, where patients were on ticagrelor [14]. The observational trials reported by Doo Sun Sim and Natsuaki [7, 8] showed 12-month follow-up outcomes, the observational trial reported by Masafumi reported 23-month follow-up outcomes [14], while the randomized trial reported by Park [11] showed 36-month follow-up outcomes, the randomized trial reported by Jeehoon Kang showed 5-year follow-up outcomes [12]. In trials between ${\mathrm{P2Y}}_{12}$ inhibitor and aspirin, no difference was observed in the proportion of patients who failed at follow-up. Table 2 (Ref. [7, 8, 11, 12, 14]) summarizes the baseline characteristics of the patients and surgeries included in our analyses. There were no significant differences in baseline data between the two groups in our analyses.

**Table 1.** **The main features of the included trials**.

	Year of publication	Region	Number of patients			${\mathrm{P2Y}}_{12\,\text{i}}$	Type of trial	Multicenter	follow-up
			Overall	Aspirin	${\mathrm{P2Y}}_{12\,\text{i}}$				
Park [11]	2016	South Korea	3243	2472	771	Clopidogrel	Observational Trial	No	36 months
Doo Sun Sim [8]	2020	South Korea	1819	1286	533	Clopidogrel	Observational Trial	Yes	12 months
Natsuaki [7]	2020	Japan	2819	1480	1339	Clopidogrel	Observational Trial	Yes	12 months
Jeehoon Kang [12]	2023	South Korea	5438	2728	2710	Clopidogrel	Randomized Trial	Yes	60 months
Masafumi Ono [14]	2022	United Kingdom	11,121	5813	5308	Ticagrelor	Randomized Trial	Yes	23 months

Abbreviation: ${\mathrm{P2Y}}_{12\,\text{i}}$, ${\mathrm{P2Y}}_{12}$ inhibitor.

**Table 2.** **Baseline clinical characteristics of patients**.

	Park 2016 [11]		Doo Sun Sim 2020 [8]		Natsuaki 2020 [7]		Jeehoon Kang 2023 [12]		Masafumi Ono 2022 [14]	
	Aspirin	${\mathrm{P2Y}}_{12\,\text{i}}$	Aspirin	${\mathrm{P2Y}}_{12\,\text{i}}$	Aspirin	${\mathrm{P2Y}}_{12\,\text{i}}$	Aspirin	${\mathrm{P2Y}}_{12\,\text{i}}$	Aspirin	${\mathrm{P2Y}}_{12\,\text{i}}$
	(n = 2472)	(n = 771)	(n = 1286)	(n = 533)	(n = 1480)	(n = 1339)	(n = 2728)	(n = 2710)	(n = 5813)	(n = 5308)
Patient Characteristics										
Mean age, y	62	64	61.1	60.9	69.7	68.1	63.3	63.3	64.1	63.7
Male (%)	73.3	73.9	78.2	78.5	73.0	79.0	75.4	74.3	77.7	77.9
Diabetes (%)	33.7	42.2	21.4	20.7	39.0	39.0	33.9	33.6	24.1	24.3
Hypertension (%)	53.2	64.5	46.0	45.7	83.0	74.0	61.3	61.4	72.8	73.4
Dyslipidemia (%)	28.5	33.5	13.3	13.2	80.0	74.0	70.6	69.5	70.4	69.6
Current smoking (%)	17.3	22.6	62.9	63.0	21.0	27.0	21.9	19.7	26.8	26.5
Chronic kidney disease (%)	8.1	10.2	NA	NA	30.0	35.0	11.9	12.9	12.2	12.2
Prior cerebrovascular accident (%)	3.2	6.1	3.2	3.1	9.2	5.3	4.8	4.2	2.2	2.4
Prior myocardial infarction (%)	19.0	18.4	2.9	2.8	17.0	14.0	15.8	16.7	22.9	21.8
Clinical presentation (%)										
Stable angina	58.9	58.0	NA	NA	67.0	62.0	28.7	27.6	55.5	51.7
UA/NSTEMI	26.5	31.3	NA	NA	NA	NA	53.7	55.2	31.6	34.7
STEMI	14.7	10.8	NA	NA	NA	NA	17.7	17.2	12.9	13.6
LVEF, %	62	62	53.3	53.5	NA	NA	NA	NA	NA	NA
Procedural Characteristics										
Angiographic disease extent										
1-vessel disease (%)	44.9	39	53.6	53.9	NA	NA	49.9	50.6	69.5	69.1
2-vessel disease (%)	33.6	38.0	30.3	30.1	NA	NA	31.3	31.4	21.8	22.7
3-vessel disease (%)	21.5	23.0	13.6	13.4	NA	NA	18.7	18.1	8.7	8.3
Target vessel location										
LM	NA	NA	1.3	1.2	1.2	2.9	4.9	5.2	2.3	2.6
LAD	NA	NA	47.4	47.6	57.0	55.0	NA	NA	52.2	50.4
LCX	NA	NA	18.7	18.4	24.0	18.0	NA	NA	31.4	31.6
RCA	NA	NA	32.6	32.8	26.0	29.0	NA	NA	36.4	37.6
Treated lesions per patient	1.0	1.0	NA	NA	1.21	1.12	1.30	1.32	1.4	1.4
No. of stents per patient	1.0	1.0	1.15	1.16	1.37	1.26	1.5	1.5	NA	NA
Maximal stent diameter, mm	3.5	3.5	3.18	3.18	NA	NA	3.08	3.08	NA	NA
Stent total length, mm	28	32	29.0	29.1	33.0	30.3	35.3	36.3	NA	NA

Data are median (25th–75th percentiles) or number of patients (%). NA means that the study didn’t present that data. Abbreviation: y, years; UA, unstable angina; NSTEMI, non ST segment elevation myocardial infarction; STEMI, acute ST segment elevation myocardial infarction; RCA, right coronary artery; LM, left main coronary artery; LAD, left anterior descending coronary artery; LCX, left circumflex coronary artery; LVEF, left ventricular ejection fraction.

**Fig. 2.**
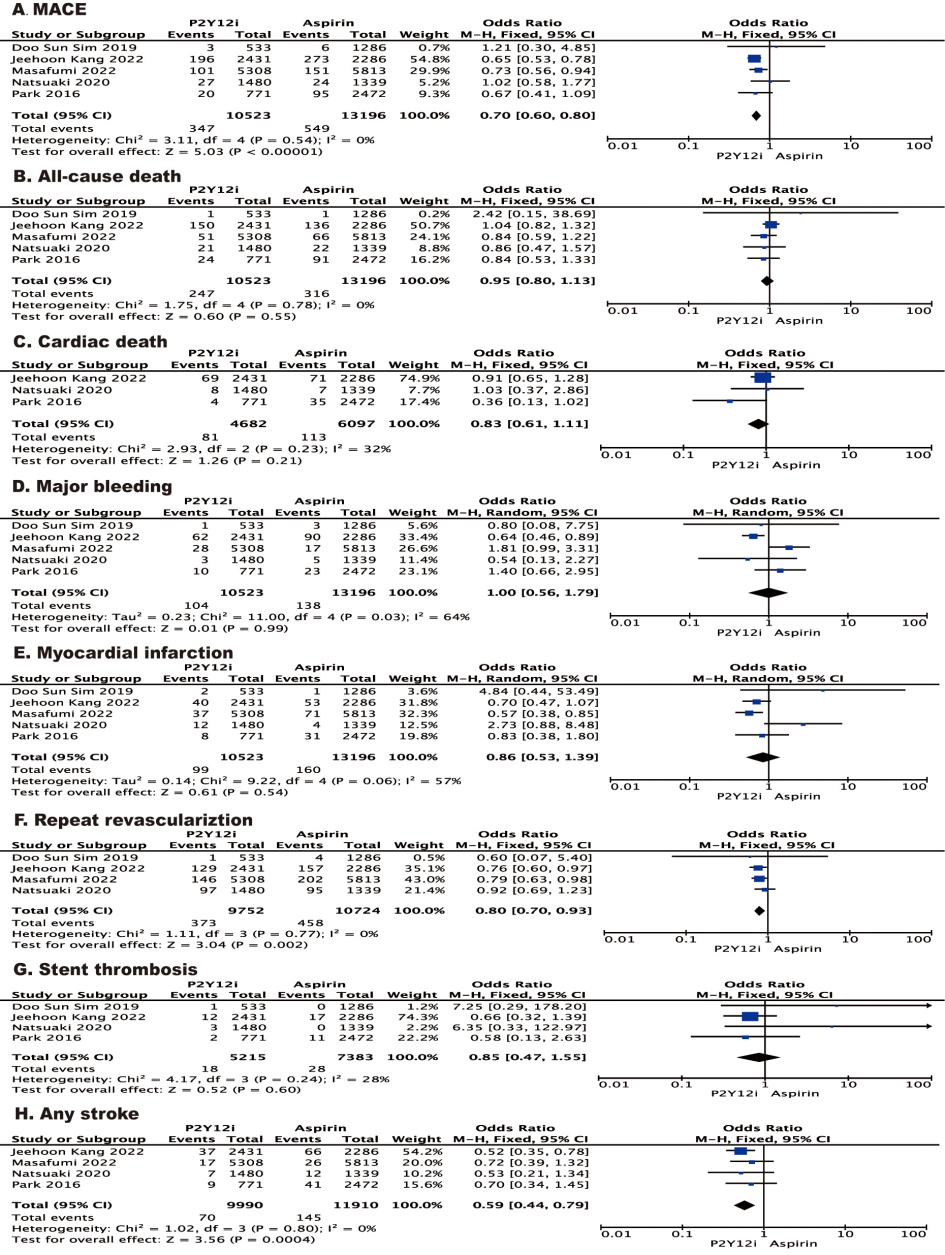
**Forest plot of the effect of ${\mathrm{P2Y}}_{12}$ inhibitor vs aspirin on the risk of outcomes for post-PCI patients after a period of DAPT. **Forest plot reporting the odds ratios of ${\mathrm{P2Y}}_{12}$ inhibitor vs aspirin: (A) MACE; (B) all-cause death; (C) cardiac death; (D) major bleeding; (E) myocardial infarction; (F) repeat revascularization; (G) stent thrombosis; (H) any stroke. PCI, percutaneous coronary intervention; MACE, major adverse cardiovascular events; DAPT, dual antiplatelet therapy.

The safety and efficacy outcomes are summarized in Fig. 2. Patients who received a ${\mathrm{P2Y}}_{12}$ inhibitor showed a risk of MACE than patients who received aspirin (odd ratio (OR) 0.70 [95% CI 0.60–0.80], $I^{2}$ = 0%, *p* < 0.00001) monotherapy following dual antiplatelet therapy 12 months after stent implantation. Specifically, the benefit of MACE in patients receiving ${\mathrm{P2Y}}_{12}$ inhibitors was primarily due to a significant reduction in repeated revascularization (OR 0.80 [95% CI 0.70–0.93], $I^{2}$ = 0%, *p* = 0.002) and any stroke (OR 0.59 [95% CI 0.44–0.79], $I^{2}$ = 0%, *p* = 0.0004). We observed no differences between patients who received aspirin and those who received a ${\mathrm{P2Y}}_{12}$ inhibitor in terms of stent thrombosis, myocardial infarction, cardiac death and all-cause death. The risk of major bleeding (OR 0.86 [95% CI 0.53–1.39], $I^{2}$ = 57%, *p* = 0.54) was similar in patients who received aspirin and those who received a ${\mathrm{P2Y}}_{12}$ inhibitor.

**Fig. 3.**
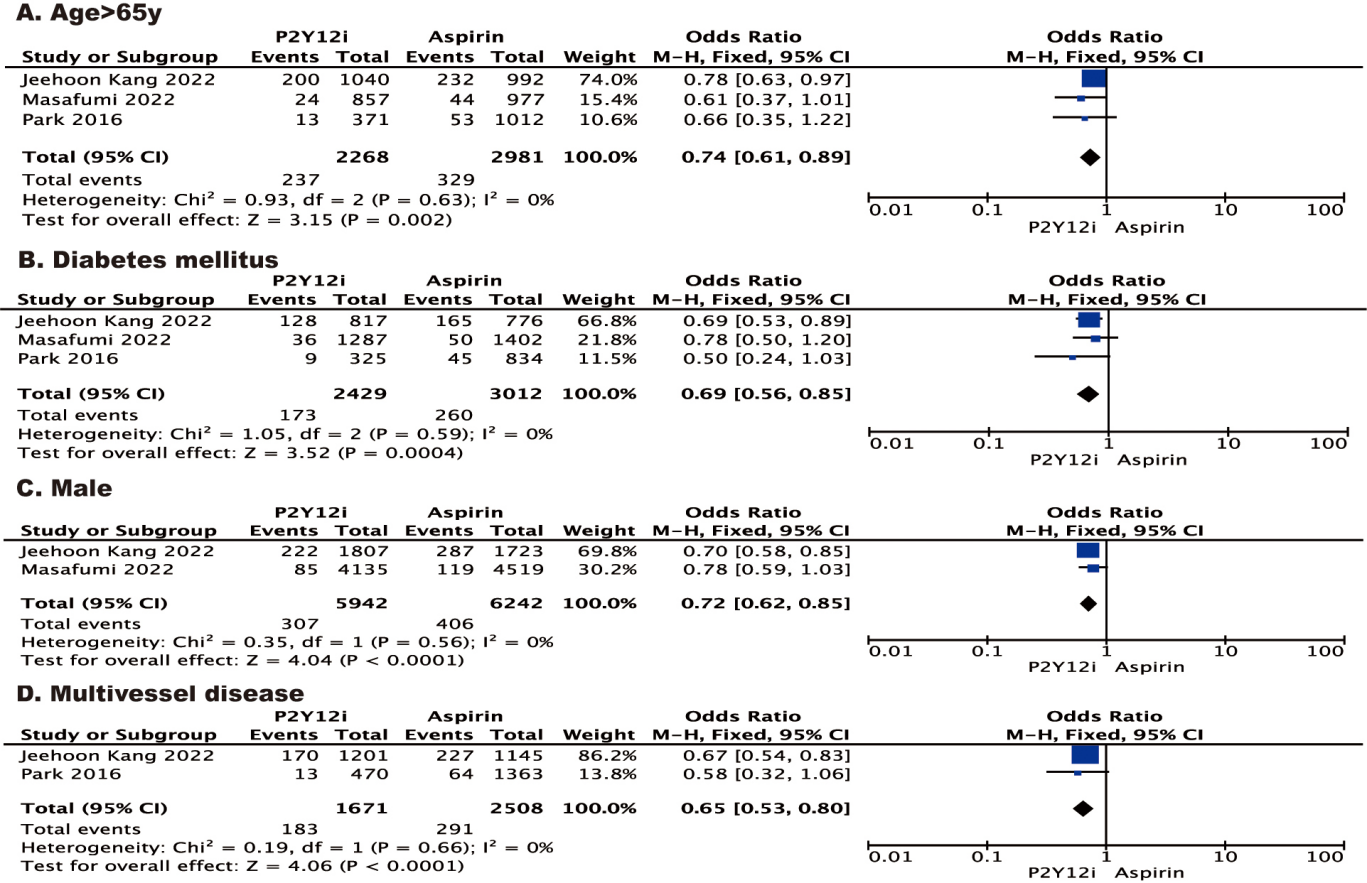
**Forest plot of stratified analysis of MACE according to the characteristics of patients.** Forest plot reporting the odds ratios of ${\mathrm{P2Y}}_{12}$ inhibitor vs aspirin: (A) age >65 y; (B) diabetes mellitus; (C) male; (D) multivessel disease. y, years.

A stratified analysis of MACE according to the characteristics of patients (i.e., age >65 years, with diabetes mellitus, male or with multivessel disease) showed results consistent with the primary analysis (Fig. 3). In another stratified analysis according to type of ${\mathrm{P2Y}}_{12}$ inhibitor, the results for MACE and death from any cause were consistent with the primary analysis, while the risk of myocardial infarction was significantly lower (OR 0.57 [95% CI 0.38–0.85], *p* = 0.005) and the risk of major bleeding was increased in patients who received ticagrelor monotherapy (OR 1.81 [95% CI 0.99–3.31], *p* = 0.05).

The results of the risk of bias assessment with the Newcastle-Ottawa Scale for cohort studies and the RoB2 of randomized control trials are presented in **Supplementary Tables 2,3**. Five studies had a lower risk of overall bias.

Stata 14.0 was used to investigate the impact of a single study on the overall pooled estimate for each predefined outcome. It was observed that deleting any of the studies did not affect the following results (**Supplementary Fig. 1**): MACE, all-cause death, repeat revascularization, stent thrombosis, myocardial infarction and any stroke. Cardio death and major bleeding may be affected by trial Jeehoon Kang [12]. Fortunately, we can find in Revman’s results that $I^{2}$ and *p* values of cardio death meet the fixed effect condition. As for the bleeding results, we will further discuss the subgroup analysis according to the different ${\mathrm{P2Y}}_{12}$ inhibitors.

## 4. Discussion

In this study, we compared monotherapy with aspirin versus a ${\mathrm{P2Y}}_{12}$ receptor inhibitor for secondary prevention in patients with ischemic heart disease after PCI following DAPT. The main findings of the present study are: (1) The risk of MACE is lower in patients receiving a ${\mathrm{P2Y}}_{12}$ receptor inhibitor compared with those receiving aspirin, which is driven by repeated revascularization and stroke. (2) Clopidogrel does not increase the risk of major bleeding, however, ticagrelor showed an increased risk of major bleeding.

Following a routine duration of DAPT, the patients may have the option of aspirin or ${\mathrm{P2Y}}_{12}$ receptor inhibitor for long-term SAPT for secondary prevention of cardiovascular events. Aspirin is classically considered the SAPT of choice following DAPT discontinuation after PCI. Notably, most randomized trials for secondary prevention assessing long-term aspirin therapy to establish its cornerstone role in the secondary prevention of cardiovascular disease were done decades ago [17]. ${\mathrm{P2Y}}_{12}$ inhibitors are the most commonly used antiplatelet drugs as an alternative to aspirin and are especially suitable for patients who are intolerant or allergic to aspirin [18, 19]. Previous studies have shown that ${\mathrm{P2Y}}_{12}$ inhibitors could at least provide similar protective effects in patients with established atherosclerosis, compared to aspirin [5]. The pharmacodynamics of ${\mathrm{P2Y}}_{12}$ inhibitors endows them with more profound platelet inhibition than aspirin [20]. Furthermore, a previous study found that clopidogrel was actually more effective than aspirin in atherosclerotic cardiovascular disease (ASCVD) secondary prevention, with a reduced risk of MACE, but with similar safety results [21]. As for more focused patients who received PCI, HOST-EXAM Extended (Harmonizing Optimal Strategy for Treatment of Coronary Artery Stenosis–Extended Antiplatelet Monotherapy) Study indicated clopidogrel monotherapy as compared with aspirin monotherapy showed lower rates of the composite net clinical outcome after PCI with drug-eluting stent (DES) [12]. Given these promising results, we conducted this meta-analysis intending to provide more evidence for the optimal long-term antiplatelet strategy after standard DAPT.

**Fig. 4.**
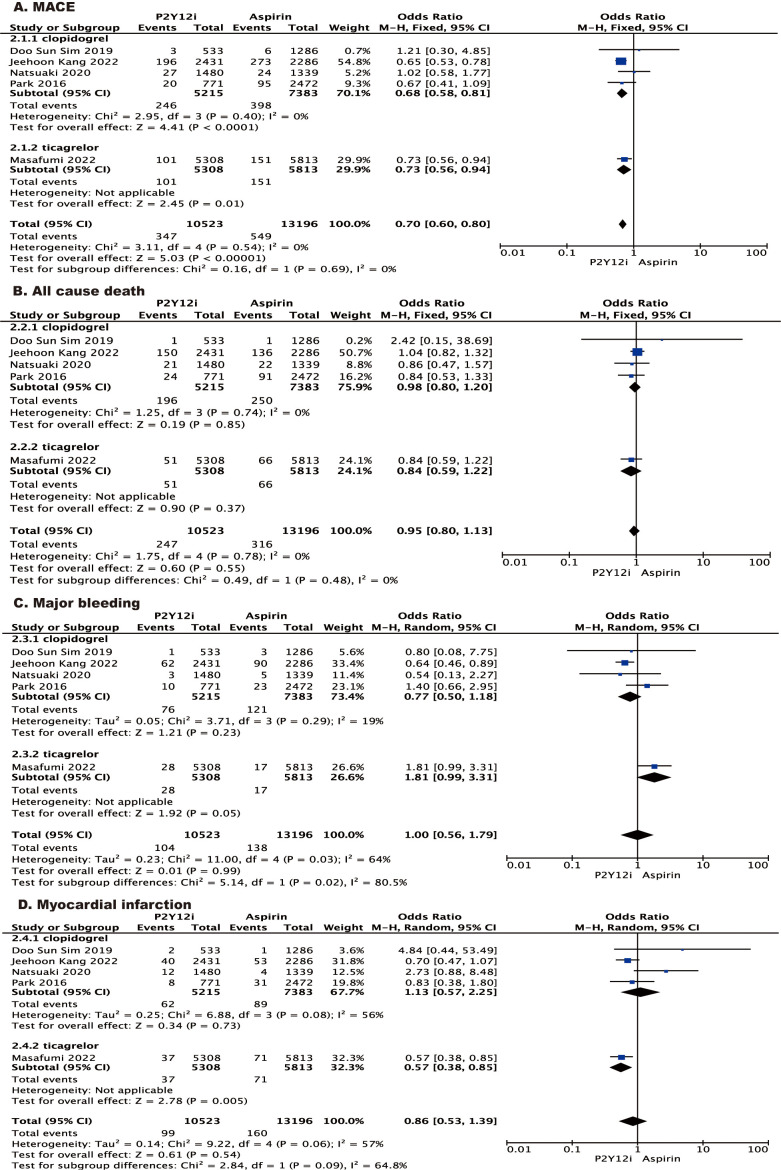
**Forest plot of stratified analysis according to type of ${\mathrm{P2Y}}_{12}$ inhibitor.** Forest plot reporting the odds ratios of ${\mathrm{P2Y}}_{12}$ inhibitor vs aspirin: (A) MACE; (B) all-cause death; (C) major bleeding; (D) myocardial infarction. MACE, major adverse cardiovascular events.

Our present meta-analysis includes 5 studies (3 observational studies and 2 RCTs), and the results indicate that ${\mathrm{P2Y}}_{12}$ inhibitor significantly reduced MACE compared to aspirin. Of note, this benefit of reduction in MACE was primarily derived from a significant reduction in repeat revascularization and any stroke. As for endpoint of all-cause death, cardiac death, myocardial infarction and stent thrombosis, no obvious benefit was observed. For the safety endpoint, the incidence of major bleeding was found to be no different between the ${\mathrm{P2Y}}_{12}$ inhibitor group and the aspirin group. These results were similar to that reported in the study of Tan *et al*. [13]. What’s different from their findings is thatwe found a reduction in the risk of repeat revascularization. This may be due to the large sample size and the longer follow-up time. To our interest, no reduction in risk of myocardial infarction was found and this was similar to previous studies [13, 17]. However, this finding differs from that in the study of Andò *et al*. [9]. What needs to be pointed out is that reduction in myocardial infarction between the two monotherapies does not convert into a decreased risk of cardiovascular death. This paradox is hard to explain. It was multifactorial and may include the influence of competing risks due to insufficient follow-up time, or variability in patient selection in the trials [22]. As for the specific type of ${\mathrm{P2Y}}_{12}$ inhibitor, ticagrelor seems more promising. Ticagrelor monotherapy was associated with a reduced risk of myocardial infarction (MI) compared to aspirin monotherapy, which is mainly derived from the results of GLOBAL LEADERS trial [14] (shown in Fig. 4). Due to the different pharmacokinetic and pharmacodynamic properties of clopidogrel and ticagrelor, ticagrelor may have more rapid and effective platelet inhibition. The PLATO (the Study of Platelet Inhibition and Patient Outcomes) trial indicated that ticagrelor proved to be superior to clopidogrel in ACS patients [23]. In theory, adequate antiplatelet therapy such as using ticagrelor would be more effective in patients with high-ischemic risk, such as patients undergoing complex PCI or those with ACS [24, 25]. For a safety endpoint, major bleeding was analyzed. Our findings found no difference in the risk of major bleeding between patients treated with ${\mathrm{P2Y}}_{12}$ inhibitors and those treated with aspirin. This is mainly because clopidogrel has a significantly lower risk of major bleeding than aspirin. Taken alone, however, patients who received ticagrelor had an increased risk of major bleeding compared to those who received aspirin (shown in Fig. 4). Therefore, this means that aspirin monotherapy may be better than ticagrelor monotherapy to avoid unnecessarily increased risk of severe bleeding, especially in those patients who are at high risk of bleeding.

There were several limitations to be mentioned. Firstly, this meta-analysis was derived from the study-level data but not individual patient-level data. This was the inherited drawback of meta-analysis. Secondly, only available data from published literature were used, while some outcomes were not reported. Of note, only one study involving the comparison between ticagrelor and aspirin could be obtained. More studies are warranted to verify the association between ${\mathrm{P2Y}}_{12}$ receptor inhibitors and their outcomes. Thirdly, the population is heterogeneous. Most studies have focused on Asian patients, while only one study was added with Ticagrelor in a European population. There were only 2 available randomized controlled trials that directly compared the two monotherapy treatments after discontinuation of DAPT after PCI, and their limited statistical power provided a theoretical basis for our meta-analysis. However, we conducted sensitivity analysis and the final results were consistent.

## 5. Conclusions

${\mathrm{P2Y}}_{12}$ inhibitor monotherapy following DAPT discontinuation after PCI showed a reduced risk for MACE, repeat revascularization and stroke compared with aspirin monotherapy. There was a similar risk for all-cause death, cardiac death and major bleeding. Our meta-analysis indicates that ${\mathrm{P2Y}}_{12}$ inhibitor monotherapy is potentially superior to aspirin for secondary prevention in the post-PCI population without an increased risk of major bleeding, but ticagrelor was associated with an increased risk of bleeding events compared to aspirin monotherapy.

## Data Availability

The data used and analyzed during the current study are available from the corresponding author on reasonable request.

## Abbreviations

PCI, percutaneous coronary intervention; post-PCI, post percutaneous coronary intervention; DAPT, dual antiplatelet therapy; MACE, major adverse cardiovascular events; CCS, chronic coronary syndrome; ACS, acute coronary syndrome; SAPT, single antiplatelet therapy; MI, myocardial infarction; RCTs, randomized controlled trials; ASCVD, Atherosclerotic Cardiovascular Disease; DES, drug eluting stent.

## Supplementary Material

Supplementary material associated with this article can be found, in the online version, at https://doi.org/10.31083/j.rcm2410284.
