# Supplementary material for: P2Y12 Inhibitor vs Aspirin Monotherapy Following Dual Antiplatelet Therapy after Percutaneous Coronary Intervention: An Updated Meta-Analysis
Source: Rev Cardiovasc Med. 2023 Oct 8;24(10):284. doi: 10.31083/j.rcm2410284 (PMC11273139; doi:10.31083/j.rcm2410284)

**Supplementary Table 1. Search strategy.**

| Electronic database | Search strategy |
| --- | --- |
| PubMed (NCBI) | ((("P2Y12"[All Fields] OR "clopidogre"[All Fields] OR "ticlopidine"[All Fields] OR "ticagrelor"[All Fields] OR "prasugrel"[All Fields] OR "thienopyridine"[All Fields] OR "antiplatelet"[All Fields]) AND ("aspirin"[All Fields] OR "acetylsalicylic acid"[All Fields]) AND ("alone"[All Fields] OR "prevention"[All Fields])) NOT "Review"[Title/Abstract]) AND (2015/1/1:2022/11/20[pdat]) |
| Embase | (‘P2Y12’:ab,ti OR ‘clopidogrel’:ab,ti OR ‘ticlopidine’:ab,ti OR 'ticagrelor':ab,ti OR 'prasugrel':ab,ti OR ‘thienopyridine’:ab,ti OR 'antiplatelet’:ab,ti) AND (‘aspirin’:ab,ti OR ‘acetylsalicylic acid’:ab,ti) AND (‘alone’:ab,ti OR ‘prevention’:ab,ti) AND (‘article’:it) |
| Cochrane | (“P2Y12” OR “clopidogrel” OR “ticlopidine” OR “ticagrelor” OR “prasugrel” OR “thienopyridine” OR “antiplatelet”) AND (“aspirin” OR “acetylsalicylic acid”) AND (“alone” OR “prevention”) |

**Supplementary Table 2. Risk of bias across individual** **randomized control trials.**

| **Num** | **Author/Year** | **Random sequence generation (selection bias)** | **Allocation concealment (selection bias)** | **Blinding of participants and personnel (performance bias)** | **Blinding of outcome assessment (detection bias)** | **Incomplete outcome data (attrition bias)** | **Selective reporting (reporting bias) Low riskUnclear riskHigh risk** | **Other bias** |
| --- | --- | --- | --- | --- | --- | --- | --- | --- |
| 1 | Jeehoon Kang 2022 | Low risk | Low risk | Low risk | Low risk | Low risk | Low risk | Unclear risk |
| 2 | Masafumi Ono 2022 | Low risk | Low risk | Low risk | Low risk | Unclear risk | Low risk | Unclear risk |

**Supplementary Table 3. Result of quality assessment using the Newcastle-Ottawa Scale for cohort studies.**

| **Num** | **Study** | **Selection** | | | | **Comparability** | **Exposure** | | | **Score** |
| --- | --- | --- | --- | --- | --- | --- | --- | --- | --- | --- |
|  |  | **Adequate definition of cases** | **Representativeness of the cases** | **Selection of controls** | **Definition of controls** | **Control for important factor ^a^** | **Ascertainment of exposure** | **Same method of ascertainment for cases** | **Non-response rate** |  |
| 1 | Park 2016 | ***** | ***** | ***** | ***** | ****** | ***** | ***** | ***** | **9** |
| 2 | Doo Sun Sim 2019 | ***** | ***** | ***** | ***** | ***** | ***** | ***** | ***** | **8** |
| 3 | Natsuaki 2020 | ***** | ***** | ***** | ***** | ****** | ***** | ***** | ***** | **9** |

^a^. A maximum of 2 stars can be allotted in this category, one for age, the other for other controlled factors.

**Supplementary Fig. 1. Sensitivity analysis.**


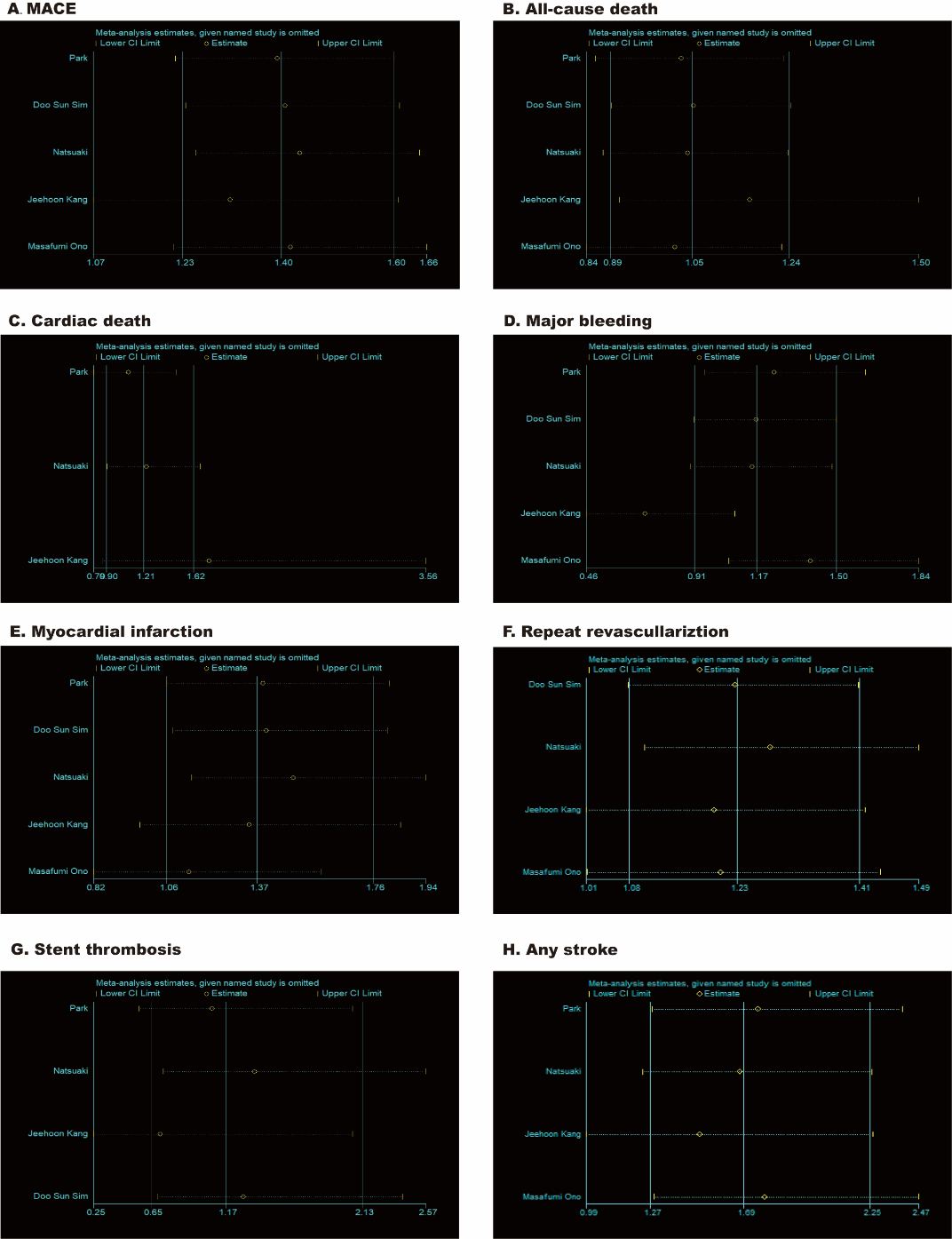

Supplement: Supplementary file 1 [file 2153-8174-24-10-284-s1.zip › Supplementary Material.docx]
